# Shielding the oil reserves: the scutellum as a source of chemical defenses

**DOI:** 10.1093/plphys/kiac038

**Published:** 2022-02-09

**Authors:** Katherine M Murphy, Elly Poretsky, Huijun Liu, Nikola Micic, Annika Nyhuis, Joerg Bohlmann, Eric A Schmelz, Philipp Zerbe, Alisa Huffaker, Nanna Bjarnholt

**Affiliations:** Donald Danforth Plant Science Center, St. Louis, Missouri 63132, USA; Section of Cell and Developmental Biology, University of California at San Diego, La Jolla, California 92161, USA; Department of Molecular Biology and Genetics, Aarhus University, Gustav Wieds Vej 10, DK-8000 Aarhus C, Denmark; Copenhagen Plant Science Center, Department of Plant and Environmental Sciences, University of Copenhagen, Frederiksberg 1871, Denmark; Plant Biochemistry Laboratory, Department of Plant and Environmental Sciences, University of Copenhagen , Frederiksberg 1871, Denmark; Copenhagen Plant Science Center, Department of Plant and Environmental Sciences, University of Copenhagen, Frederiksberg 1871, Denmark; Plant Biochemistry Laboratory, Department of Plant and Environmental Sciences, University of Copenhagen , Frederiksberg 1871, Denmark; Bruker Daltonik GmbH & Co. KG, Bremen 28359, Germany; Michael Smith Laboratories, University of British Columbia, Vancouver, British Columbia V6T 1Z4, Canada; Section of Cell and Developmental Biology, University of California at San Diego, La Jolla, California 92161, USA; Department of Plant Biology, University of California Davis, One Shields Avenue, Davis, California 95616, USA; Section of Cell and Developmental Biology, University of California at San Diego, La Jolla, California 92161, USA; Copenhagen Plant Science Center, Department of Plant and Environmental Sciences, University of Copenhagen, Frederiksberg 1871, Denmark; Plant Biochemistry Laboratory, Department of Plant and Environmental Sciences, University of Copenhagen , Frederiksberg 1871, Denmark

## Abstract

The cereal scutellum is a hub for diverse specialized defense metabolism and pathway discovery.

Dear Editor,

The scutellum of monocotyledonous plants is synonymous with the first non-foliar leaf or cotyledon (Kiesselbach, 1999). In germinating monocot seeds, scutella function in absorbing nutrients from the endosperm, transferring sugars and amino acids to the developing plant, and providing oil reserves (Huang, 1992). This critical role as a nutrient conduit also makes the scutellum prone to pest and pathogen attack. Grain crop scutella have long served as a model for studying lipid body (López-Ribera et al., 2014) and gibberellin (GA) phytohormone formation (Betts et al., 2020) during seed development and germination, but its role as a source of defense metabolites for protecting valuable nutrient reserves of the germinating seed has been widely overlooked. Here, we highlight recent insights into the specialized defense metabolism in cereal scutella.

Until reaching autotrophy, nutrient-rich germinating seedlings depend upon finite carbon reserves while being exposed in moist soil environments to pathogens and herbivores. As a terminal, fully differentiated tissue, the scutellum of a germinating monocot seedling functions both in general metabolism and as a source of chemical defense. In contrast to dicots, monocot scutella remain below ground and are thereby exposed to environments with high moisture, complex microbiomes, and higher disease potential. The specialized metabolism of the scutellum in monocots has been best studied in maize (*Zea mays*) and sorghum (*Sorghum bicolor*). Recent work highlighted this tissue as an exceptionally rich source of diverse specialized metabolites that mediate biotic stress protection (Huang and Backhouse, 2004; Huffaker et al., 2011; Schmelz et al., 2011; Christensen et al., 2016; Ding et al., 2017, 2019, 2020; Montini et al., 2020). Beyond maize and sorghum, germinating oat (*Avena sativa*) and barley (*Hordeum vulgare*) scutella accumulate unique defense-related phenolamide derivatives termed avenanthramides (Matsukawa et al., 2000) and hordatine glycosides, respectively (Gorzolka et al., 2014).

Biotic stress protection in maize and sorghum seedlings largely relies on benzoxazinoids (BX) and cyanogenic glycosides, respectively (Ding et al., 2020; Montini et al., 2020). These defense metabolites are rapidly activated by β-glucosidases following cellular damage. Fungal elicitation of foliar maize tissues suppresses BX biosynthesis and instead induces diverse sesquiterpenoid, diterpenoid, and flavonoid pathways (Ding et al., 2019, 2020; Förster et al., 2021). Studies with fungal-infected tissues and scutella in maize seedlings (Mellon and West, 1979) led to the discovery of a diverse array of antibiotic metabolites, including sesquiterpenoid zealexins and α/β-costic acids (Huffaker et al., 2011; Ding et al., 2017, 2020), diterpenoid kauralexins (Schmelz et al., 2011; Christensen et al., 2018; Ding et al., 2019) and dolabralexins (Mafu et al., 2018), as well as 9-lipoxygenase derived oxylipins termed death acids (Christensen et al., 2015, 2016; Figure 1, A–C). Interestingly, while these defense metabolites are not commonly found in healthy maize tissues, they are present in the seedling scutellum. Likewise, sorghum scutella produce the cyanogenic glucoside dhurrin and related compounds constitutively (Montini et al., 2020), while the 3-deoxyanthocyanidin phytoalexins apigeninidin and luteolinidin are biosynthesized following *Fusarium* infection of this tissue (Huang and Backhouse, 2004). Similarly to dhurrin and its derivatives in sorghum, death acids and BX (Cambier et al., 2000) in maize are largely absent from dry seed, but the two first were found to accumulate in the scutella during and after germination (Christensen et al., 2016; Montini et al., 2020), and thus appear to be synthesized de novo in the scutellum during germination and seedling development. The presence and production of complex defenses in seedling grain crop scutella presents an opportunity for discoveries of biochemical pathways that underlie plant–microbe and plant–herbivore interactions (Ding et al., 2021).

**Figure 1:**
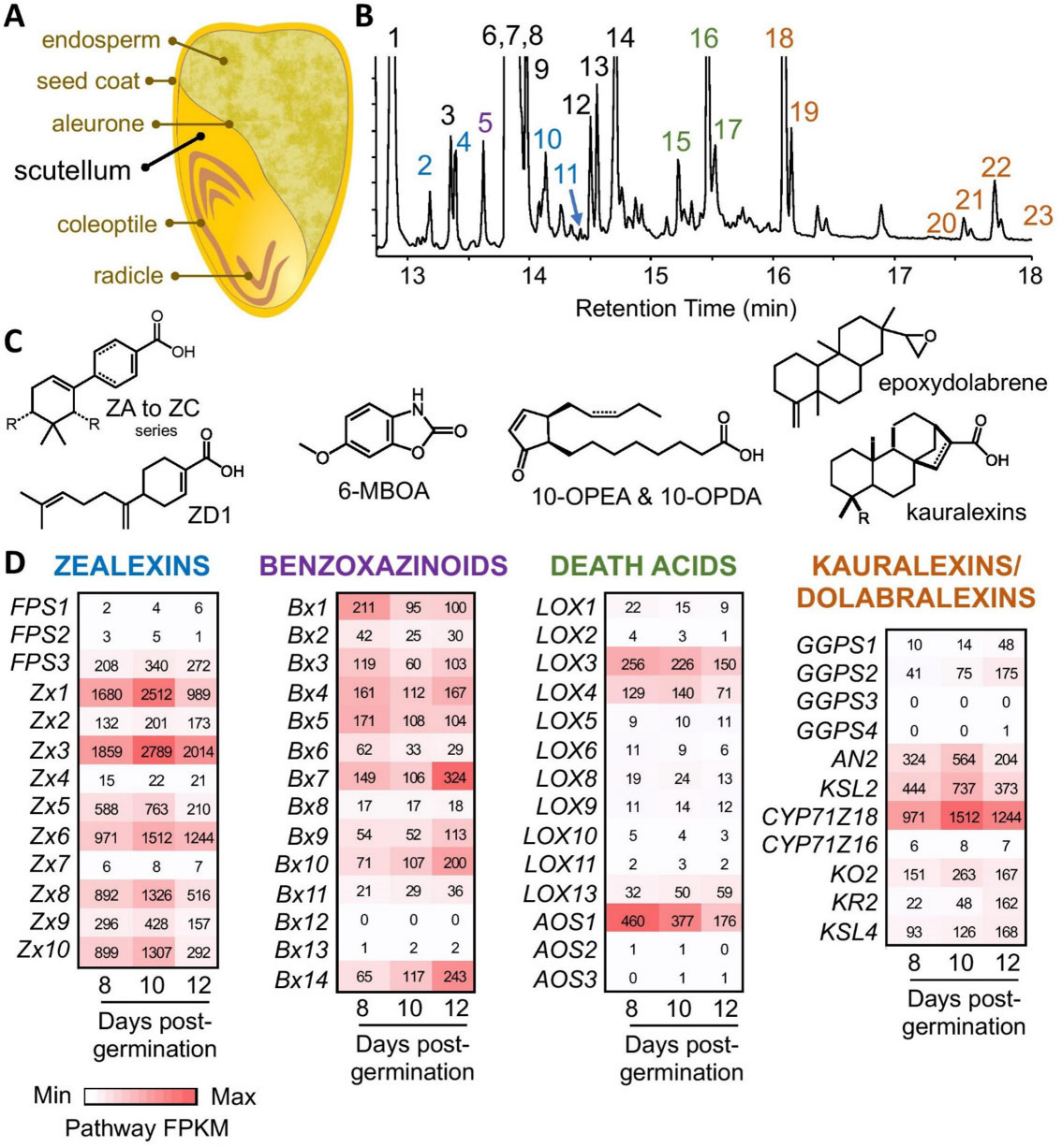
After germination, maize scutella biosynthesize a diverse array of protective specialized metabolites. A, Cross section diagram of a maize seed prior to germination highlighting the large oil-rich scutellum. B, Gas chromatography (GC) mass spectrometry (MS) (GC/MS) total ion chromatographic (TIC) profile of metabolites (predominantly methyl ester derivatives) present in maize scutellum 12 d after germination in soil. Fatty acids (*black* numbers) detected include (1) palmitic acid, (3) heptadecanoic acid, (6) oleic acid, (7) steric acid, (8) linoleic acid, (9) linolenic acid, (12) oleic acid propyl ester, (13) linoleic acid propyl ester, (14) cis-13-eicosenoic acid. Propyl esters are likely formed during tissue extraction in acidic 1-propanol. Sesquiterpenoid antibiotics in the zealexin (ZX) biosynthetic pathway (*blue* numbers) include (2) ZD1, (4) ZA1, (10) ZB1, and (11) ZA2. The BX degradation product (*purple* number) of 2,4-dihydroxy-7-methoxy-1,4-benzoxazin-3-one (DIMBOA) is (5) 6-methoxy-2-benzoxazolinone (6-MOBA). Maize 9-lipoxygenase derived oxylipins, termed death acids, detected (*green* numbers) are (15) trans-10-oxo-11-phytoenoic acid (10-OPEA), (16) cis-10-OPEA, and (17) cis-10-oxo-11-phytodienoic acid (10-OPDA). Diterpenoid antibiotics in the kauralexin pathway (*orange* numbers) include (18) KA1, (19) KB1, (20) KA2, (21) KB2, (22) KA3, and (23) KB3. C, Representative structures of defense metabolites present in post-germination scutellum include ZX (A–D series), BX, death acids, kauralexins, and dolabralexins. Dolabralexins are present in scutellum tissues but exist at low abundance, not readily visible in a TIC. D, Heatmaps of transcript levels in FPKM (fragments per kilobase of transcript per million mapped reads) for specialized biosynthetic pathway genes in the Ky21 scutellum at 8, 10, and 12 d post germination in soil. Corresponding gene IDs and related information (B73 RefGen V4) are in Supplemental Table 2. *Zx6*/*Cyp71Z18* and *Zx7*/*Cyp71Z16* are synonymous and shared between sesquiterpenoid (ZX) and diterpenoid (kauralexin and dolabralexin) pathways.

To better understand defense-related biochemical pathways in maize scutella (Figure 1A), we performed parallel metabolite and RNA-Seq analyses (Supplemental Figure S1 and Supplemental Tables S1 and S2, respectively) with the Ky21 inbred line at 8, 10, and 12 d after planting seeds into soil. In the scutella, we found a diverse array of acidic zealexins, BX derivatives such as 6-methoxy-benzoxazolin-2-one (6-MBOA), cyclopentenone death acids, and acidic kauralexins (Figure 1B), that co-occurred with fatty acids at levels sufficient to be detected in a total ion chromatogram. Quantified at days 6, 8, 10, and 12, the scutella show a steady increase in zealexin (3.8-fold), kauralexin (8.4-fold), and death acid (115-fold) pathway products and intermediates, yet a 50% decrease in a BX marker 6-MBOA (Supplemental Figure S1). These results are consistent with the hypothesis that dynamic processes of de novo synthesis and catabolism co-occur in post-germination scutella. Multiple biosynthetic pathways for maize defenses have been described in the context of fungal-elicited stem and root tissues (Mafu et al., 2018; Zhou et al., 2018; Ding et al., 2019, 2020); however, our RNA-Seq analysis demonstrates that transcripts encoding enzymes in these pathways are also highly expressed in seedling scutella (Figure 1D). In contrast to fungal-elicited maize stems, in which zealexin, α/β-costic, and kauralexin biosynthetic pathways are strongly activated while the BX pathway is suppressed (Ding et al., 2020), transcripts underlying BX biosynthesis remain elevated in post-germination scutella simultaneous with activation of terpenoid antibiotic pathways (Figure 1 and Supplemental Table S2). Metabolite analyses suggest a small decrease in the BX derivative 6-MBOA (Supplemental Figure S1); however, it is likely that BX glucoside (undetectable by GC/MS) biosynthesis continues in the scutella well after germination. Collectively, post-germination maize scutella display an extensive array of concomitantly active defense pathways uncommonly observed together in healthy seedling tissues. The observed accumulation of terpenoid defense metabolites in scutella may also shape maize–microbe interactions beyond protection against potential pathogens. Because primary root development is driven by the scutellum in maize, and emergence of the primary root initiates rhizosphere microbiome establishment, terpenoids in the scutellum may influence early establishment and/or composition of the root microbiome (Ding et al., 2020; Murphy et al., 2021).

In studies with sorghum seeds, we previously used matrix-assisted laser desorption/ionization-mass spectrometry imaging (MALDI-MSI) to show highly dynamic changes of dhurrin and dhurrin derivatives, known as recycling products, during germination (Montini et al., 2020). Like dhurrin, these recycling products accumulated in the scutellum shortly after imbibition as glucosides of *p*-hydroxymandelic acid and *p*-hydroxyphenylacetic acid, compounds that may have anti-microbial or signaling functions (Kope et al., 1991; Montini et al., 2020; YujiaLiu et al., 2021). These compounds may shape the early root microbiome and they may also act as signaling compounds alongside with known scutellum phytohormones (Betts et al., 2020). To further explore the biochemical defense arsenal in sorghum scutella at the very early stages of germination and seedling development, we analyzed samples corresponding to those previously published (Montini et al., 2020), using the recently developed laser post-ionization MALDI-MSI (MALDI-2-MSI) technology for enhanced sensitivity toward low abundance compounds (Soltwisch et al., 2020). Dhurrin (Figure 2D; embryonic axis) and two of its recycling products (Figure 2, E and F; embryonic axis and scutellum) were detected in the same tissues as previously reported by Montini et al. (2020). Here, we confirmed compound identities by comparison with authentic standards in extract analysis and the fact that all compounds were absent in extracts and MS-images of a sorghum line mutated in the dhurrin biosynthetic pathway. The remaining compounds in Figure 2 are putatively identified based on *m*/*z* values and deduced sum formulae, partially supported by isotope ratios (see Supplemental Methods and Supplemental Table S3), serving to show the potential of the technology for the detection of biochemicals localized to the scutella. Complete identification requires, for example, extract analysis and, ideally, availability of authentic standards. This would also allow utilization of the ion mobility MS feature of the instrument, which can furthermore reveal possible presence and differential distribution of isobaric compounds. Some MSI instruments can perform MS/MS; however, for the majority of compounds visualized in Figure 2, the low signal intensities would render this impossible. Sorghum seeds accumulate a wide variety of polyphenols (Xiong et al., 2020), including many compounds containing hydroxycinnamic acids. We found several metabolites with *m*/*z* values and deduced sum formulae matching such compounds accumulated in both embryo and scutellum, in addition to an apparent phospholipid that was preferentially localized to the scutellum (Figure 2). Two compounds (Figure 2, F and H) were detected as their [M + H]^+^ ions, which is unusual in MALDI-MSI, but prevalent in MALDI-2-MSI. The increase in ion abundance afforded by MALDI-2 in the positive mode is usually mainly comprised of [M + H]^+^ ions, whereas signal intensities of [M + Na]^+^ and [M + K]^+^ ions are less or not at all increased (Soltwisch et al., 2020). As the compounds shown in the MS images in Figure 2, F and H were not detected as their sodiated or potassiated ions, they would therefore likely have remained undetected in a standard MALDI-MSI analysis.

**Figure 2:**
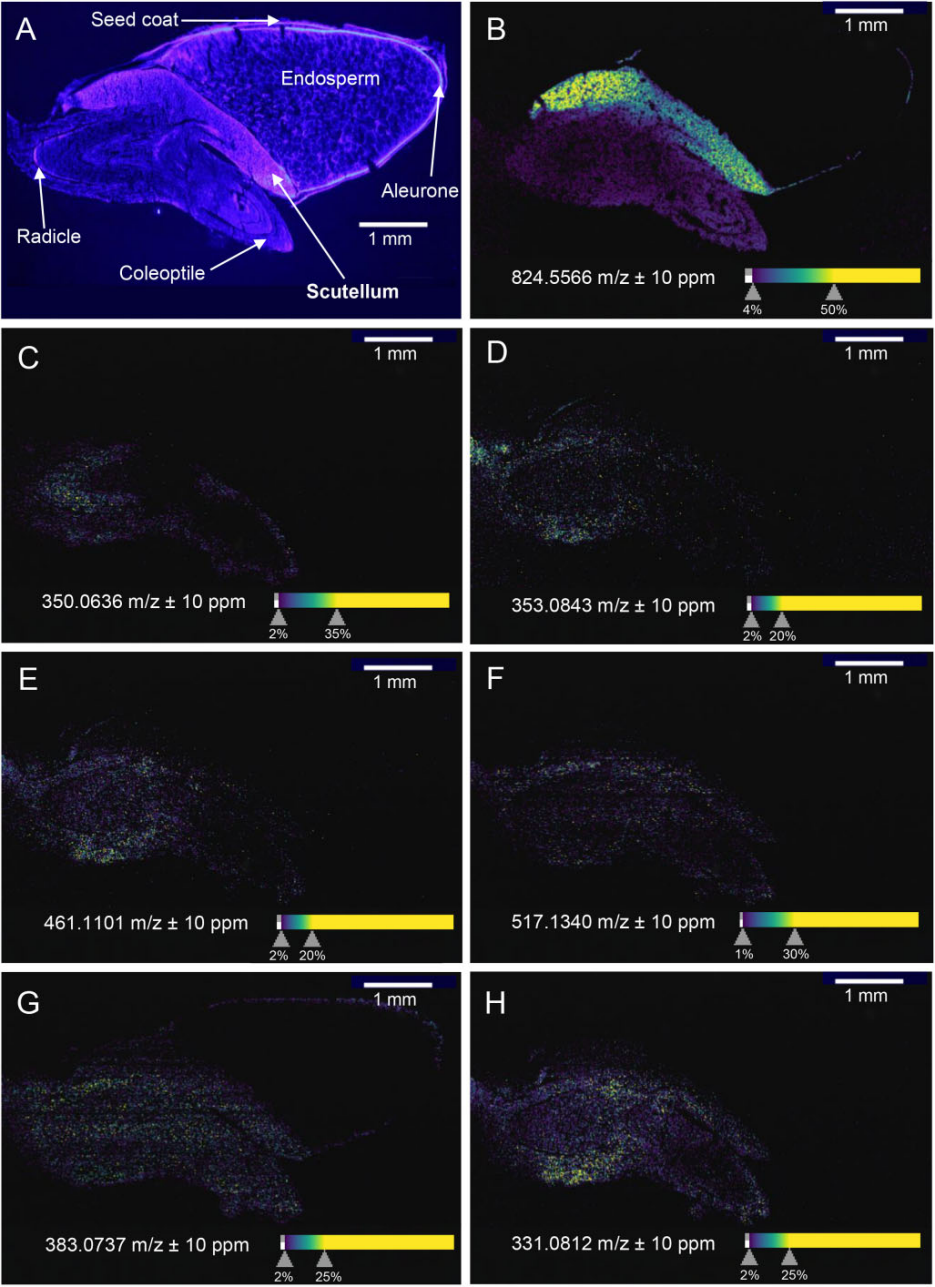
Distribution of metabolites in germinating sorghum seeds. Cross sections of germinating sorghum seed were analyzed by timsTOF fleX MALDI-2 in positive ion mode at 10 µm lateral resolution to obtain images of molecular distributions across the samples. All images A–H are displayed with scale bars for size. A, Fluorescence image of cross section for reference. B, [C_44_H_84_NO_8_P+K]^+^, sum formula corresponding to a putative phospholipid, 1,2-dioleoyl-sn-glycero-3-phosphocholine, or 1-linoleoyl-2-stearoyl-sn-glycero-3-phosphocholine. C–E, Dhurrin and two derivatives previously demonstrated to accumulate in scutellum and/or embryonic axis (radicle + coleoptile) (Montini et al., 2020). C, Dhurrin, detected as [M+K]^+^, only in the embryonic axis. D, Dhurrin acid, detected as [M+Na]^+^, in scutellum and embryonic axis. E, Glutathione derivative of dhurrin, GS-*p*-hydroxyphenylacetic acid, detected as [M+Na]^+^, in scutellum and embryonic axis. F–H, Phenolic compounds potentially containing caffeic acid moieties, accumulated in scutellum and other tissues. Sum formulae deduced from *m*/*z* values, but absolute structures not confirmed. F, [C_25_H_24_O_12_+H]^+^, sum formula corresponding to 3,4-dicaffeoylquinic acid, apparently more highly accumulated in scutellum than in embryonic axis. G, [C_18_H_16_O_8_+Na]^+^, sum formula corresponding to rosmarinic acid, but most likely an isomer as this compound is not known to accumulate in Poaceae. Detected in all living tissues. H, [C_17_H_14_O_7_+H]^+^. Dhurrin and dhurrin acid can be found conjugated with caffeic acid in sorghum. This deduced sum formula corresponds to a hypothetical caffeic acid conjugated *p*-hydroxymandelic acid (dhurrin acid aglycone), but may more likely be a flavanone or flavone such as dimethylquercetin. Detected in scutellum and embryonic axis. All MS images: For increased clarity, low- and high-end intensity thresholds have been adjusted. The corresponding color-coded scale bars are displayed under each mass spectrometry image.

Collectively, our recent and present findings across different grain crops highlight the scutellum as an epicenter of diverse seedling defense metabolism. While the unique arrays of specialized metabolites and their specific roles in defense or signaling will differ between species, the recent work featured in this letter showcases how MS-based metabolomics and MALDI- and MALDI-2-MS imaging together with transcriptome- and genome-based discovery provide tissue-specific insights into the largely overlooked specialized metabolism of the scutellum. At the seedling stage, scutella can be leveraged as a discovery hub for protective biosynthetic pathways. Approaches such as single-cell RNA sequencing and precision gene editing may offer additional powerful tools to understand and ultimately leverage scutellum specialized metabolism for crop protection. Effects of the maternal genotype on scutellum chemistry have not yet been investigated, as most studies have utilized defined inbreds, and present further questions for exploration. The modest size, yet extensive biochemical diversity of the scutellum creates a highly amenable target tissue to investigate functions of specialized metabolism in plant–biotic interactions at a vulnerable stage of crop development.

## Accession number

SRA archive submission number BioProjectID: PRJNA750086.

## Supplemental data

**Supplemental Figure S1.** Changes in abundance of specialized metabolites in germinating maize scutella are consistent with de novo biosynthesis in multiple pathways.

**Supplemental Table S1.** Gene expression quantification using RNA-Seq data of maize Ky21 scutellum tissue at 8, 10, and 12 d post germination for all genes.

**Supplemental Table S2.** Gene expression data (FPKM) of selected maize biosynthetic pathway genes in Ky21 scutellum tissue at 8, 10, and 12 d post-germination.

**Supplemental Table S3.** Theoretical and measured isotope ratios for putative compounds from Figure 2.

**Supplemental Methods.** Preparation of maize scutella RNA-Seq data and analysis of maize seed tissue gene expression, GC/MS analysis, and MALDI-MSI analysis.

## Supplementary Material

kiac038_Supplementary_Data

## Notes

K.M.M. and E.P. contributed equally. K.M.M., J.B., E.S., P.Z., A.H., and N.B. wrote the letter. E.P., P.Z., J.B., E.S., and A.H. carried out the maize experiments and data analysis for Figure 1. For Figure 2, H.L., N.M., and A.N. prepared the sorghum samples. A.N. performed the MALDI-2-MSI analysis and A.N. and N.M. the data analysis. All authors read and commented on the letter.

The author responsible for distribution of materials integral to the findings presented in this article in accordance with the policy described in the Instructions for Authors (https://academic.oup.com/plphys/pages/General-Instructions) is Nanna Bjarnholt (nnb@plen.ku.dk).
