# Supplementary material for: Shielding the oil reserves: the scutellum as a source of chemical defenses
Source: Plant Physiol. 2022 Feb 9;188(4):1944–9. doi: 10.1093/plphys/kiac038 (PMC8968280; doi:10.1093/plphys/kiac038)
Supplement: kiac038_Supplementary_Data [file kiac038_supplementary_data.zip › Scutellum_Review_TableS3.docx]

**Shielding the oil reserves: the scutellum as a source of chemical defenses**

Katherine M. Murphy, Elly Poretsky, Huijun Liu, Nikola Micic, Annika Nyhuis, Joerg Bohlmann, Eric Schmelz, Philipp Zerbe, Alisa Huffaker, Nanna Bjarnholt

**Table S3. Theoretical and measured isotope ratios for putative compounds from Figure 2.**

| **Ion** | **m/z** | **Theoretical** | **Measured** |
| --- | --- | --- | --- |
| [C_44_H_84_NO_8_P+K]^+^ (Figure 2B) | | | |
| M | 824.5566 ± 10 ppm | 100 | 100 |
| M+1 | 825.5600 ± 13 ppm | 39 | 49 |
| M+2 | 826.5602 ± 10 ppm | 18 | 21 |
| [C_25_H_24_O_12_+H]^+^ (Figure 2F) | | | |
| M | 517.1341 ± 10 ppm | 100 | 100 |
| M+1 | 518.1375 ± 13 ppm | 41 | 28 |
| M+2 | 519.1398 ± 10 ppm | 18 | 6 |
| [C_18_H_16_O_8_+Na]^+^ (Figure 2G) | | | |
| M | 383.0737 ± 10 ppm | 100 | 100 |
| M+1 | 384.0771 ± 13 ppm | 15 | 20 |
| M+2 | 385.0793 ± 10 ppm | 3 | 4 |
| [C_17_H_14_O_7_+H]^+^ (Figure 2H) | | | |
| M | 331.0812 ± 10 ppm | 100 | 100 |
| M+1 | 332.0846 ± 10 ppm | 12 | 19 |
| M+2 | 333.0868 ± 10 ppm | 2 | 3 |
