# Supplementary material for: Shielding the oil reserves: the scutellum as a source of chemical defenses
Source: Plant Physiol. 2022 Feb 9;188(4):1944–9. doi: 10.1093/plphys/kiac038 (PMC8968280; doi:10.1093/plphys/kiac038)
Supplement: kiac038_Supplementary_Data [file kiac038_supplementary_data.zip › Scutellum_SuppMethods_revised.docx]

**Shielding the oil reserves: the scutellum as a source of chemical defenses**

Katherine M. Murphy^a^*, Elly Poretsky^b^*, Huijun Liu^c,d,e^, Nikola Micic^d,e^, Annika Nyhuis^f^, Joerg Bohlmann^g^, Eric Schmelz^b^, Philipp Zerbe^h^, Alisa Huffaker^b^, Nanna Bjarnholt^d,e,†^

**Supplemental Methods**

**Preparation of maize scutella RNA-Seq data and analysis of maize seed tissue gene expression**

Using the maize inbred line Ky21, total RNA was isolated from 150 mg scutella tissues harvested 8, 10, and 12 days post-planting using previously reported methods (Kolosova et al., 2004). Scutella tissue from 8 plants per time point was pooled to generate uniform average samples. RNA integrity and concentration were verified using Agilent Bioanalyzer 2100 RNA Nano chip assays. Construction of non-normalized cDNA libraries and subsequent transcriptome sequencing was performed at the McGill University and Génome Québec Innovation Centre (Montreal, QC, Canada). Preparation of cDNA libraries was performed from 10 μg of total RNA using the Illumina mRNA Seq Sample Preparation Kit, with normalization to 100 ng total mRNA for each sample. Yields and correct fragment sizes were assessed using an Agilent High Sensitivity DNA chip. Illumina HiSeq 2000 sequencing was performed by multiplexing three cDNA libraries per lane using 7 pmol of each library and 100 bp pair-ended runs. Raw data is available in the Sequence Read Archive (BioProjectID is PRJNA750086). Raw paired-end reads were cleaned using Trimmomatic (Bolger et al. 2014, v0.39, with the parameters LEADING:3 TRAILING:3 SLIDINGWINDOW:4:15 MINLEN:36 HEADCROP:12). Cleaned reads were aligned to the B73 V4 reference genome (ensemble 4.44) using the STAR aligner (Dobin et al. 2013, v2.7.7a, with the parameters outFilterMismatchNoverLmax 0.04 alignIntronMax 6000) and sorted using Sambamba (Tarasov et al. 2015, v0.8.0, default parameters). Mapped reads were quantified using featureCounts (Liao et al. 2014, v1.6.4, default parameters) and converted to FPKM expression data.

**GC/MS analysis**

GC/MS analysis of maize seedling scutella follows from previously used methods employing tissue extraction with acidic 1-propanol, organic solvent partitioning, trimethylsilyldiazomethane methylation of the carboxylic acids, sample enrichment using vapor phase extraction and chromatographic separation on a DB-35 column (Ding et al., 2019; Ding et al., 2020). For metabolite quantification (Supplemental Fig. S1) we employed isobutane-chemical

ionization-GC/MS and a U-^13^C-linolenic acid internal standard as described (Ding et al., 2020).

**MALDI-MSI analysis**

Sorghum grain (var. BTx 623) were imbibed for 6 h and germinated for 18 h on sterilized wet filter paper, all in the dark at 25 °C. The germinated grain were snap frozen, embedded in carboxymethylcellulose, sectioned longitudinally at 10 µm using a cryotome and freeze dried as previously described (Montini et al., 2020). A fluorescence image of the cross section was acquired on a Nikon ECLIPSE Ni-U Fluorescence microscope with UV filter (EX 361-389 (BP), 415 (LP), EM 430-490 (BP)) using a Canon EOS1200D camera and Scientific Acquisition Software. The scale bar was added using the software ImageJ. Matrix was applied using a TM3 sprayer (HTX technologies). Twenty passes of a 2,5-dihydroxyacetophenone (2,5-DHAP) matrix solution (15 mg/mL in 80 % ACN:10 % MeOH:10 % H_2_O + 0.1 % TFA) were sprayed using a nozzle temperature of 70 °C, a flow rate of 0.125 mL/min and a N_2_ gas pressure of 68.9 kPa.

Imaging data was acquired on a timsTOF fleX MALDI-2 instrument (Bruker Daltonics GmbH & Co. KG, Bremen, Germany), allowing for laser-based post-ionization. The instrument was operated in positive ion mode, and ions were accumulated from 28 laser shots per pixel in the mass range of m/z 300-1500. A “single” laser spot without active beam scan with an approximate spot diameter of 5 µm was used. For imaging, a raster width of 10 µm was used. The experiment was conducted in MALDI-2 mode using a trigger delay of 10 µs and a laser frequency of 1 kHz. Data analysis was performed with SCiLS^TM^ Lab 2021b software from Bruker Daltonics, and images in Fig. 2 were plotted with 10 ppm accuracy. The identities of dhurrin, dhurrin acid and GS-pOHACN were previously confirmed by Montini et al. (2020). The remaining compounds are tentatively identified from their deduced sum formulae, partially supported by isotope ratios as shown in Supplemental Table S3 and explained in the following: Most compounds were detected at very low signal intensities. In such cases, as MSI analysis does not involve chromatographic separation, isotope ratios are very easily skewed if isotope ions fall under the detection limit or by signal contributions from noise and possible overlaps with other compounds present in the same area of the tissue. E.g. the m/z value of the M+3 ion of the compound in Figure 2H happens to overlap with that of sodiated dhurrin, which was likely present at low abundance. However, when we selected areas with approximately 50 pixels of the highest possible intensity of each ion (using the SCiLS^TM^ Lab 2021b software), the M+1 and M+2 ions roughly matched the theoretical values, which were calculated using IsotopePattern Version 3.0 (Build 200.7.23) software from Bruker Daltonik. These ratios are shown below. The proposed phospholipid in Figure 2B was detected at somewhat high signal intensity and therefore matches the theoretical values better than some of the other compounds, although M+1 is a little low.

**SRA archive submission number** BioProjectID: PRJNA750086

**Supplemental References**

Bolger AM, Lohse M, Usadel B (2014) Trimmomatic: a flexible trimmer for Illumina sequence data. Bioinformatics 30: 2114–2120

Ding Y, Murphy KM, Poretsky E, Mafu S, Yang B, Char SN, Christensen SA, Saldivar E, Wu M, Wang Q, et al (2019) Multiple genes recruited from hormone pathways partition maize diterpenoid defences. Nature Plants 5: 1043–1056

Ding Y, Weckwerth PR, Poretsky E, Murphy KM, Sims J, Saldivar E, Christensen SA, Char SN, Yang B, Tong A-D, et al (2020) Genetic elucidation of interconnected antibiotic pathways mediating maize innate immunity. Nat Plants 6: 1375–1388

Dobin A, Davis CA, Schlesinger F, Drenkow J, Zaleski C, Jha S, Batut P, Chaisson M, Gingeras TR (2013) STAR: ultrafast universal RNA-seq aligner. Bioinformatics 29: 15–21

Kolosova N, Miller B, Ralph S, Ellis BE, Douglas C, Ritland K, Bohlmann J (2004) Isolation of high-quality RNA from gymnosperm and angiosperm trees. Biotechniques 36: 821–824

Liao Y, Smyth GK, Shi W (2014) featureCounts: an efficient general purpose program for assigning sequence reads to genomic features. Bioinformatics 30: 923–930

Montini L, Crocoll C, Gleadow RM, Motawia MS, Janfelt C, Bjarnholt N (2020) Matrix-Assisted Laser Desorption/Ionization-Mass Spectrometry Imaging of Metabolites during Sorghum Germination. Plant Physiol 183: 925–942

Tarasov A, Vilella AJ, Cuppen E, Nijman IJ, Prins P (2015) Sambamba: fast processing of NGS alignment formats. Bioinformatics 31: 2032–2034
